# Net return distributions when metaphylaxis is used to control bovine respiratory disease in high health-risk cattle

**DOI:** 10.1093/tas/txaa020

**Published:** 2020-02-17

**Authors:** Elliott J Dennis, Ted C Schroeder, David G Renter

**Affiliations:** 1 Department of Agricultural Economics, University of Nebraska – Lincoln, Lincoln, NE; 2 Department of Agricultural Economics, Kansas State University, Manhattan, KS; 3 Center for Outcomes Research and Epidemiology, College of Veterinary Medicine, Kansas State University, Manhattan, KS

**Keywords:** antimicrobial, bovine respiratory disease, cattle, economic simulation, metaphylaxis, net returns

## Abstract

This study’s objective was to estimate net returns and return risk for antimicrobial metaphylaxis options to manage bovine respiratory disease (BRD) in high health-risk feedlot cattle. The effectiveness of antimicrobials for metaphylaxis varies by cattle population. How differing antimicrobial effectiveness translates to net return profitability for heterogeneous cattle populations is less understood. Net returns and return risk were assessed using a net return simulation model adapted to allow for heterogeneity in high health-risk cattle placement characteristics and antimicrobial choice to control BRD. The net return model incorporated how antimicrobials modify BRD health and performance outcomes. Health and performance outcomes were calibrated from published literature and proprietary feedlot data. Proprietary data came from 10 Midwestern feedlots representing nearly 6 million animals and 50,000 cohorts. Twelve placement-by-metaphylaxis decision combinations were assessed: high health-risk steer placement demographics were 600 or 800 lb steers placed in Winter (Oct–Mar) or Summer (Apr–Sept) managed with one of three different health programs: “no metaphylaxis,” “Upper Tier” antimicrobial, or “Lower Tier” antimicrobial. Net return distributions were compared between “no metaphylaxis” and a specific antimicrobial tier within specific cattle populations. We found the expected incremental net return of administering an “Upper Tier” (“Lower Tier”) antimicrobial for metaphylaxis compared to “no metaphylaxis” for high health-risk steers was $122.55 per head ($65.72) for 600 lb and $148.65 per head ($79.65) for 800 lb winter placements. The incremental expected net return and risk mitigated by metaphylaxis varied by placement weight, season, and antimicrobial choice. The probability net returns would decline by at least $50 per head was significantly reduced (from approximately 4% to 40%) when any antimicrobial was used on high health-risk steers. Both tiers of antimicrobials used for metaphylaxis increased expected net returns and decreased net return variability relative to no metaphylaxis. Thus, feedlots were more certain and realize a greater profit on high health-risk pens of steers when metaphylaxis was used. This occurred because the reduction in cattle health and performance outcomes using any antimicrobial was sufficiently large to cover added initial and subsequent antimicrobial costs. Results aid in assessing metaphylaxis strategies in high health-risk cattle.

## INTRODUCTION

Antimicrobials used to improve the well-being, health, and performance of cattle arriving at feedlots have received considerable public attention. Metaphylaxis, administration of an antimicrobial, generally via injection, is used by 39% of U.S. feedlots with 1000+ head capacity selectively on 17% of cattle to reduce adverse effects of bovine respiratory disease (BRD) in high health-risk cattle (United States Department of Agriculture, 2019). Randomized control trials have generally confirmed metaphylaxis can reduce morbidity and mortality in feedlot cattle where health-risk susceptibility is high (O’Connor et al., 2013; Abell et al., 2017). Categorization as high health risk generally refers to cattle with one or more risk factors for BRD, which may include unknown health history, recent weaning, and various source and transport stressors (Nickell and White, 2010; Ives and Richeson, 2015; Smith et al., 2017).

When cohorts of cattle arrive at feedlots, feedlots assess animal health risks and decide whether to manage cattle with metaphylaxis. Perceived benefits of metaphylaxis for reducing cattle morbidity and mortality are weighed against costs to process, treat, and monitor cattle during their time on feed. If metaphylaxis is elected, feedlots must select the type of antimicrobial to administer (Nickell and White, 2010). The selection of the specific antimicrobial to use is primarily based on veterinary consultation, past experience, and duration of action (United States Department of Agriculture, 2013). While the effectiveness and cost of antimicrobials used for metaphylaxis vary, how these differences translate into expected net return distributions for heterogeneous cattle has not been described (DeDonder and Apley, 2015; Ives and Richeson, 2015).

Realized cattle morbidity and mortality conditional on antimicrobial choice and administration are unknown until after cattle harvest. As such, animal health outcomes are uncertain when the metaphylaxis decision is made. Stochastic net return simulations have been used to value BRD for dairy, cow-calf, and feedlot cattle (Van der Fels-Klerx et al., 2001; Buhman et al., 2003; Nor et al., 2012; Theurer et al., 2015; Wang et al., 2018) and to value animal health protocols such as metaphylaxis in feedlot cattle (Dennis et al., 2018). Stochastic simulations are distinctly different than deterministic simulations because they incorporate uncertainty via probability distributions obtained from historical data, expert opinion, and/or published literature. However, no studies have examined how net returns differ by the type of antimicrobial used for metaphylaxis across different cattle placement demographics.

The objective of this study is to measure net return and return risk between “Upper Tier” and “Lower Tier” antimicrobials used for metaphylaxis and a “no metaphylaxis” option in high health-risk feedlot cattle. In particular, we test whether expected net return distributions vary across cattle placement weight, placement season, and antimicrobial tier administered.

## MATERIALS AND METHODS

Cattle feeding net returns vary across management, marketing, and animal health protocols. Cattle feeding net return distributions are estimated using a variation of the stochastic simulation model developed by Dennis et al. (2018). The primary purpose of Dennis et al. (2018) was to estimate the economic value of metaphylaxis to the U.S. fed cattle industry. This economic value was then used to determine how removing metaphylaxis as an animal health protocol would impact feedlots, processors, and consumers. In order to calculate the overall industry-level impact of metaphylaxis, the authors used an “average” animal gender, placement season, and antimicrobial used. We modify their simulation method by allowing placement season, weight, and antimicrobial used for metaphylaxis to vary. In what follows, we briefly describe the cattle feeding economic decision framework used in our stochastic simulation emphasizing how we incorporate heterogeneity into the Dennis et al. (2018) cattle feeding net return simulation model.

### Cattle Feeding Net Return Stochastic Simulation

We consider four types of high health-risk steers purchased by feedlots—two cattle placement weights (600 or 800 lb) and two placement seasons (Oct–Mar referred to as “Winter” or Apr–Sept referred to as “Summer”). In our simulation, we considered three different health management options for high health-risk steers: “no metaphylaxis,” “Upper Tier” antimicrobial, or “Lower Tier” antimicrobial. High health-risk cattle are cattle that are classified to be at-risk for BRD and should be managed with metaphylaxis. Antimicrobials were categorized into “Upper” and “Lower” tiers based on categorizations from the work of Abell et al. (2017) where they classified antimicrobials into tiers conditional on odds ratio (OR) confidence intervals for different morbidity and mortality outcomes. Thus, our simulation compared eight different metaphylaxis scenarios (one gender, two placement weights, two seasons, and two tiers of antimicrobials used for metaphylaxis) to four “no metaphylaxis” scenarios (one gender, two placement weights, two seasons).

Regardless of initial health status and health management strategy, cattle can become sick and/or die. Feedlots realize final morbidity and mortality only at cattle harvest. In our model, all cattle, regardless of initial health-risk status, possessing clinical signs of BRD are pulled and treated. Sick cattle incur greater health costs (HC ↑), gain less weight per day during feeding (ADG ↓), and require more feed to gain an additional pound of weight (AFC ↑) (Tennant et al., 2014). Feedlots do not sell dead animals (CSW = 0) losing the initial cost of the feeder (FDRC) plus any cost for yardage (YC ≥ 0), feed (FC ≥ 0), interest (IC ≥ 0), and health treatments (HC ≥ 0). Morbidity and mortality combine to increase total feeding costs (TC ↑) effectively decreasing profit (π ↓). Mortality poses a high cost to feedlots and is the primary driver of net return and return risk in our stochastic simulation.

After initial processing, cattle that survive and reach an expected harvest weight are sold on a live-weight basis. Thus, feedlots choose the type of cattle to purchase, initial and supplemental health protocols, and cattle target finish weight. Given this, cattle feeding net returns (π) per head for a given cohort of cattle can be specified as (Dennis et al., 2018):

$$\begin{array}{l} \;\pi \;=\underset{Total\;revenue}{\underbrace{TR}}-\underset{Total\;costs}{\underbrace{FDRC-YC-FC-HC-IC}} \end{array}$$ (1)

$$\begin{array}{l} \underset{Total\;revenue}{\underbrace{TR}}=\underset{Fed\;Price}{\underbrace{FP}}\;\underset{Total\;costs}{\underbrace{\begin{array}{l} \times \;\{\underset{Finshed\;weight}{\underbrace{CSW}}\\ \times \;\underset{Transportation\;weight\;loss}{\underbrace{(1-SHRINK)}}\\ \times \underset{Lbs.\;of\;animals\;solds}{\underbrace{(1-MORT-ICLL)\;\}\;}}) \end{array}}}\\ +\underset{Revenue\;from\;culled\;animals}{\underbrace{(CULL\;\times \;CULLW\;\times \;CULLP)}} \end{array}$$ (2)

$$\begin{array}{l} \underset{Cost\;\;\;to\;\;\;purchase\;\;\;cattle}{\underbrace{FDRC}}=\underset{Feeder\;Price}{\underbrace{FRP}}\;\times \;\underset{Placement\;\;\;weight\;\;\;of\;\;\;feeder}{\underbrace{CPW}} \end{array}$$ (3)

$$\begin{array}{l} \underset{Yardage\;\;\;costs}{\underbrace{YC}}=\underset{Fixed\;\;\;Rate}{\underbrace{0.30}}\;\times \;\underset{Days\;\;\;on\;\;\;feed}{\underbrace{DOF}} \end{array}$$ (4)

$$\begin{array}{l} \underset{Feed\;costs}{\underbrace{FC}}=\underset{Price\;of\;corn}{\underbrace{FEED}}\;\begin{array}{l} \times \underbrace{\{\underset{Feed\;conversion}{\underbrace{AFC}}\;}\\ \times \;\underset{\underbrace{\;Total\;weight\;gain\;while\;at\;feedlot\;}}{\underbrace{\left[CSW\times (1-MORT-CULL\right)-CPW\;]\;\}\;}} \end{array}\\ Total\;amount\;of\;feed\;consumed\;while\;at\;feedlot \end{array}$$ (5)

$$\begin{array}{ll} \underset{Interest\;costs}{\underbrace{IC}}= & \underset{Entire\;feeder\;and\;half\;of\;all\;other\;costs}{\underbrace{\left\{0.5\;\times \;\left[YC+FC+HC\right]+FDRC\right\}}}\;\\ & \times \;\underset{Interest\;rate}{\underbrace{\left(IR/365\right)}}\;\\ & \times \;\underset{Days\;on\;feed}{\underbrace{DOF}} \end{array}$$ (6)

Table 1 describes each variable in detail.

**Table 1.** Feeding net return variables

Variables	Description	Value/Calculation
Simulated		
ADG	Average daily gain during feeding (lb/head/day)	See Table 3
AFC	Average pounds of feed consumed per pound of weight gain (lb feed/lb gain)	See Table 3
DOF	Number of days on feed (days)	
FC	Feed cost ($/head)	See Eq. 5
HC	Animal health care cost including metaphylaxis, pull-and-treat, vaccinations, labor costs, etc. ($/head)	See Table 3
IC	Interest cost ($/head)	See Eq. 6
MORT	Proportion of mortality in purchased group	See Figure 1
TR	Total revenue from cattle sales ($/head)	See Eq. 2
YC	Yardage cost of feeding cattle ($/head)	See Eq. 4
π	Net feeding returns ($/head)	See Eq. 1
Fixed		
CPW	Cattle purchase weight (lb/head)	600, 800
CSW	Finished animal weight (lb/head) if animal reaches maturity	1,350
CULL	Proportion chronically ill animals culled from the remaining cohort	0.0140
CULLP	Price received for culled animals ($/lb)	0.75 × FP
CULLW	Average weight of culled animals (lb/head)	861
FDRC	Feeder cattle purchase cost ($/head)	See Eq. 3
FEED	Corn price when cattle are placed on feed ($/lb)	0.0923
FP	Fed cattle sale price ($/lb)	1.48
FRP	Purchase price for CPW 600 (spring, winter) 800(spring, winter) lb ($/lb)	2.1461, 2.1379, 1.7591, 1.7436
IR	Annualized interest rate	0.05
SHRINK	Proportion shrink in live weight when marketed	0.04

Our objective was to determine how net return distributions change for high health-risk cattle as feedlots use different antimicrobial tiers across different placement weights and seasons. To do this, we calibrated the expected return in the simulation models by using breakeven (π = 0) feeder cattle purchase prices for the “Upper Tier” antimicrobial used for each season and placement weight. This enabled us to compare net return distributions with and without metaphylaxis and across tiers of antimicrobials used for metaphylaxis but only within its respective season and placement weight *not* across different seasons or placement weights.

### Data

Ten large commercial feedlot operations located in several Midwestern states provided two animal health and performance data sets used in this study. Cohorts are the common aggregate unit in commercial feedlot production systems. Cohort-level animal health treatment information is the primary difference between the two data sets. We define cohorts (lots or pens) as animals purchased, assembled, and managed as an observable unit. When finished cattle are marketed, closeouts records record cohort-level animal performance and health information.

Observational data used in this study included a large panel data set comprising 48,341 cohorts of cattle (about 6 million head) placed on feed during 1989–2008. This data set consisted of typical closeout information including HCs after feeding began excluding costs of metaphylaxis. These data were used to calibrate animal feeding performance over time (i.e., ADG and AFC), which varied by season, location, and animal weight. The second observational data used comprised 1,357 cohorts of cattle (about 264,000 head) placed on feed during 2014–2015. This more detailed, but smaller data set, documented both cohort and individual animal antimicrobial treatments associated with BRD enabling us to estimate the cost of metaphylaxis and facilitated our stochastic simulation around cattle mortality. Table 2 displays summary statistics for the feedlot data.

**Table 2.** Feedlot performance summary characteristics for two periods

	Mean	SD	Min	Max
January 1989–December 2008^a^				
Feed conversion (lb feed/lb gain)	6.07	0.59	3.01	9.91
Average daily gain (lb gain/day)	2.96	0.56	1.51	5.98
Mortality (%, MORT ×100)	1.24	1.76	0.00	25.64
Placement weight (lb)	683.7	128.99	304.20	1,100.00
Days on feed (days)	154.5	44.17	128.00	229.00
Gender	Steer	45.7%	Heifer	54.3%
Season	Spring	25.1%	Summer	27.4%
	Fall	24.3%	Winter	23.2%
August 2014–December 2015^b^				
Feed conversion (lb feed/lb gain)	6.16	0.58	4.30	8.76
Average daily gain (lb gain/day)	3.24	0.49	1.65	5.18
Mortality (%)	2.52	3.19	0.00	26.78
Placement weight (lb)	700.8	177.49	301.00	1096.00
Days on feed (days)	192.8	67.20	87.00	443.30
Gender	Steer	55.0%	Heifer	45.0%
Season	Spring	25.8%	Summer	23.2%
	Fall	25.8%	Winter	25.2%

^a^Period one has 48,341 cohorts/pen.

^b^Period two has 1,357 cohorts/pen.

Source: Proprietary feedlot data.

### Cattle Morbidity and Mortality

Metaphylaxis is expected to reduce morbidity and mortality in high health-risk feedlot cattle, but expected effectiveness varies by cattle placement weight, placement season, gender, and antimicrobial used (Nickell and White, 2010; DeDonder and Apley, 2015; Ives and Richeson, 2015). In our model, the impact of metaphylaxis on morbidity is reflected by its effects on three cattle performance parameters—average daily gain (ADG), average feed conversion (AFC), and HCs.

Multivariate Tobit, ordinary least squares, maximum likelihood, and linear mixed models (LMMs) have been used to model variation in ADG, veterinary/medication costs, and feed conversion in cattle across seasons, placement weights, etc. (Miller et al., 2005; Irsik et al., 2006; Belasco, 2008; Belasco et al., 2009; Dennis et al., 2018). In our study, cattle performance parameters were quantified using an LMM with cohort size (i.e., the number of head per lot), year, and feedlot specific random effects to account for the hierarchical nature of cattle feeding data where cohorts of cattle are nested within feedlots. Fixed effects for the estimation of ADG and AFC included the percentage of cohort level mortality (MORT × 100), the natural log of cattle placement weight (lnCPW), whether the cohort were steers (STEER), and whether cattle were placed between October and March (WINTER). Cohort-level HCs are estimated in a similar format to ADG and AFC but WINTER is omitted because of the short time horizon of the data. In addition, binary variables indicating the antimicrobial tier given for metaphylaxis to a cohort are included (UPPER TIER or LOWER TIER). We estimated ADG and AFC equations using the large panel data set and associated HC using the more recent detailed data set.

We conditioned cattle mortality on cattle placement weight, placement season, and antimicrobial used for metaphylaxis. Mortality distributions are known to be right-skewed with long tails, approximated using a log-normal, (zero-inflated) negative binomial or a (zero-inflated) Poisson distribution (Babcock, 2010). We modeled all mortality distributions as log-normal. Mortality distributions for the “Upper Tier” and “Lower Tier” antimicrobials were fit using the mean and standard deviation of mortality observed from proprietary production level data. However, the mortality of high health-risk cattle not managed with metaphylaxis was not observed in feedlot data because feedlots give metaphylaxis to all cattle categorized as high health risk upon arrival. Thus, mortality distributions for high health-risk cattle not treated with metaphylaxis were approximated using ORs from a mixed treatment comparison (MTC) meta-analysis (Abell et al., 2017). An MTC meta-analysis summarizes published randomized control antimicrobial trials for BRD-related cattle morbidity and mortality and can be used to assess indirect comparisons across different antimicrobials used for metaphylaxis (O’Connor et al., 2013; Abell et al., 2017).

### Differences Across Antimicrobials Used for Metaphylaxis

One important concern is the endogenous producer choice to match the type of antimicrobial used for metaphylaxis to different cattle populations. The potential endogenous decision implies that simply comparing mortality outcomes in observational data between two different antimicrobial tiers is incorrect. Even after statistically matching cattle that received different tiers of antimicrobials based on observable feedlot and pen characteristics, the antimicrobial choice is still nonrandom. Thus, in this context, traditional quasi-experimental methods to ascertain causality are insufficient. We solve this issue by developing a counterfactual mortality distribution that answers the following question: “Given we observe the mortality rate for a ‘Lower Tier’ antimicrobial, what would have been the observed mortality on that same cohort of cattle had an ‘Upper Tier’ antimicrobial been used?”

To answer this proposed counterfactual question, we first multiplied the estimated OR (0.62) for Tilmicosin (the “Lower Tier” antimicrobial) from the work of Abell et al. (2017) by a proposed hypothetical “no metaphylaxis” mortality rate. This resulted in a hypothetical proposed “Lower Tier” antimicrobial mortality rate. We then iterated through different hypothetical proposed “no metaphylaxis” mortality values until the hypothetical proposed “Lower Tier” mortality rate matched the rate observed in the more recent intensive data. Then, the corresponding “Upper Tier” antimicrobial mortality was obtained by multiplying the resulting “no metaphylaxis” mortality rate by the estimated OR (0.16) for Tulathromycin (the “Upper Tier” antimicrobial) from the work of Abell et al. (2017). Our proposed method is loosely similar to the Bayesian method used by Abell et al. (2017) to obtain differences in ORs for different types of antimicrobials. Comparing the ORs obtained using our proposed method and those obtained by Abell et al. (2017) and find that they are not statistically different from each other. Thus, we applied our procedure for each of the four different placement weight by placement season scenarios: 600 lb summer, 600 lb winter, 800 lb summer, and 800 lb winter. Thus, by using this procedure resulting mortality rates, and subsequent net return distributions, could be compared within season and placement weights. The code for this optimization method can be obtained by contacting the authors.

### Simulation to Obtain Net Return Distributions

Our simulation method closely follows the method proposed by Dennis et al. (2018) to obtain net returns. We briefly describe those methods here and a more complete and detailed description can be found in the work of Dennis et al. (2018). The simulation method is as follows. First, we specified the high health-risk steer placement weight and placement season. Second, we selected what antimicrobial tier was given, if any. Third, given these choices, we randomly selected a mortality rate from the corresponding mortality distribution. Fourth, the steer placement, metaphylaxis decision, and random mortality draw were multiplied by estimated beta coefficients from the LMM. Fifth, these stochastic ADG, AFC, and HC were used to calculate cattle feeding net return profit (*π*) from Eq. 1 for one cohort of cattle. Net return distributions for the 12 different scenarios (one gender, two placement weights, two placement seasons, three metaphylaxis options) were generated by repeating steps 1 to 5 using 5,000 Halton draws.

## RESULTS

### Descriptive Statistics

On average (min, max) cattle were placed at approximately 700 lb, gained 3 lb per day (1.5, 6.0), and had feed conversion of about 6 (3.0, 9.9) over 155 days on feed (128, 229). Cattle were placed evenly across seasons. The average mortality for period one cattle was 1.24% (0.0, 25.6). A large variation in mortality in both time periods was due to initial health status (high vs. low health risk), differing cattle populations (light vs. heavy weight), and placement season (winter vs. summer).

### Estimated Morbidity

Table 3 displays parameter estimates for the estimated ADG, AFC, and HC models. Increased mortality was associated with lower daily gains (ADG ↓), increased feed conversion (AFC ↑), and increased HCs (HC ↑). Higher placement weights were associated with higher feed conversions (AFC ↑) and higher daily gains (ADG ↓). Placement weight was excluded from the HCs equation. Cattle placed during winter months had marginally higher daily gain and higher feed conversion. If an “Upper Tier” (“Lower Tier”) antimicrobial was used for metaphylaxis, feedlots incurred an estimated $28.61 ($23.97) per head cost for administration. Company random effects suggested significant variation in company animal HCs.

**Table 3.** LMM estimation for cattle performance that serves as a proxy for cattle morbidity

	ADG	AFC	HC
Fixed effects			
Constant	–4.536 (0.10)^a^	–1.129 (0.14)	5.936 (7.80)
Mortality (MORT × 100)	–0.059 (0.00)	0.050 (0.00)	1.541 (0.80)
Log placement weight (lnCPW)	1.147 (0.01)	1.144 (0.02)	
Steer (STEER)	0.238 (0.00)	–0.272 (0.01)	1.782 (0.42)
Oct–Mar (WINTER)	0.009 (0.00)	0.004 (0.01)	
Antimicrobial (Upper Tier)			28.605 (0.85)
Antimicrobial (Lower Tier)			23.969 (0.84)
Random effects^b^			
Company	0.016	0.016	14.417
Cohort size	0.002	0.003	0.501
Placement year	0.007	0.072	
Observations	48,341	48,341	1,357
REML convergence	41,678	75,791	9,334

^a^Numbers in parenthesis () are standard errors.

^b^Variances are reported for each random effect.

Source: Author’s calculations.

### Mortality Distributions

**Figure 1.**
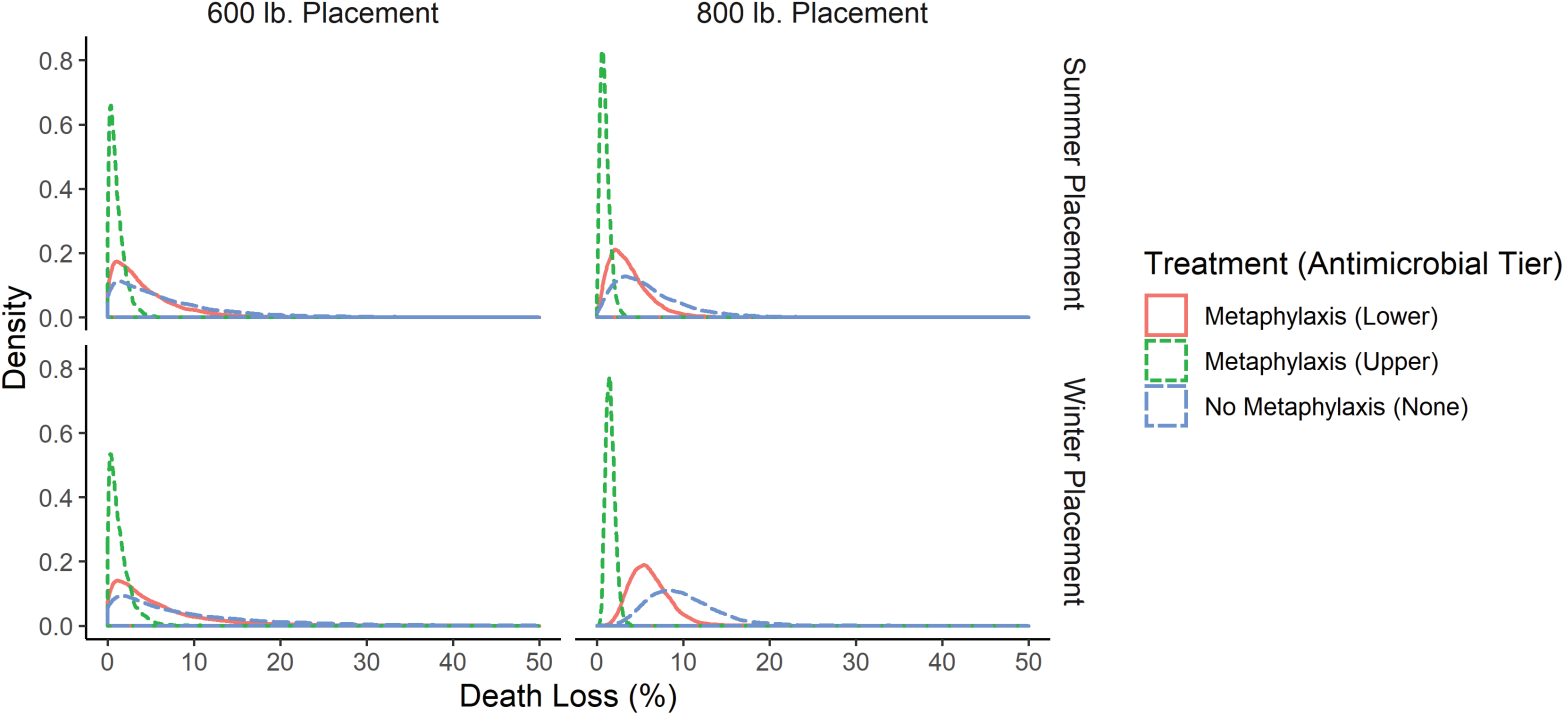
Mortality distributions for high health-risk steers by placement weight, placement season, and type of antimicrobial used for metaphylaxis. Antimicrobials used for metaphylaxis are categorized into “Upper” and “Lower” tiers based on Abell et al. (2017) who categorized them based on odds ratio (OR) confidence intervals. Source: Abell et al. (2017) and author’s calculations.

Figure 1 displays log-normal mortality distributions for high health-risk steers conditional on placement weight, season, and antimicrobial used for metaphylaxis. Both tiers of antimicrobials used for metaphylaxis reduced expected mortality and the associated variance. Table 4 displays the parameters used to calculate the log-normal mortality distributions. Winter placements, across both weights, had larger mortality mean and variances. “Upper Tier” antimicrobials on average reduced mortality by more than “Lower Tier” antimicrobials and narrowed the associated variance. Not treating with metaphylaxis resulted in larger average mortality and greater variation across all seasons and placement weights for high health-risk steers not managed with metaphylaxis. Thus, metaphylaxis reduced mortality in high health-risk steers but varied by a class of antimicrobial used.

**Table 4.** Mortality distributional assumptions for cattle type and antimicrobial treatment

Antimicrobial	Placement weight (lb)	Season	Mean^a^ (%)	SD^a^ (%)
Upper Tier	600	Summer	1.07	0.91
Upper Tier	600	Winter	1.36	1.23
Upper Tier	800	Summer	0.94	0.55
Upper Tier	800	Winter	1.57	0.54
Lower Tier	600	Summer	4.14	3.77
Lower Tier	600	Winter	5.26	5.08
Lower Tier	800	Summer	3.66	2.30
Lower Tier	800	Winter	6.10	2.24
No Metaphylaxis	600	Summer	6.68	6.49
No Metaphylaxis	600	Winter	8.49	8.75
No Metaphylaxis	800	Summer	5.90	3.96
No Metaphylaxis	800	Winter	9.83	3.87

^a^To account for endogenous producer decisions in using specific antimicrobials on specific cattle populations we use the odds ratios from Abell et al. (2017) for the lower tier antimicrobial and the mortality observed in lower tier antimicrobials to solve for the mortality of the control. We then use this control mortality and the odds ratios for the upper tier antimicrobial to obtain the mortality for the upper tier antimicrobial. This allows us to obtain the mortality of different antimicrobials on different cattle populations. A similar producer was used to find the standard deviations. In some cases, due to the absence of sufficient observations of steer pens (i.e., n ≥ 20), a pooled steer and heifer estimate was used.

Source: Proprietary feedlot data and Abell et al. (2017).

### Net Return Distributions

**Figure 2.**
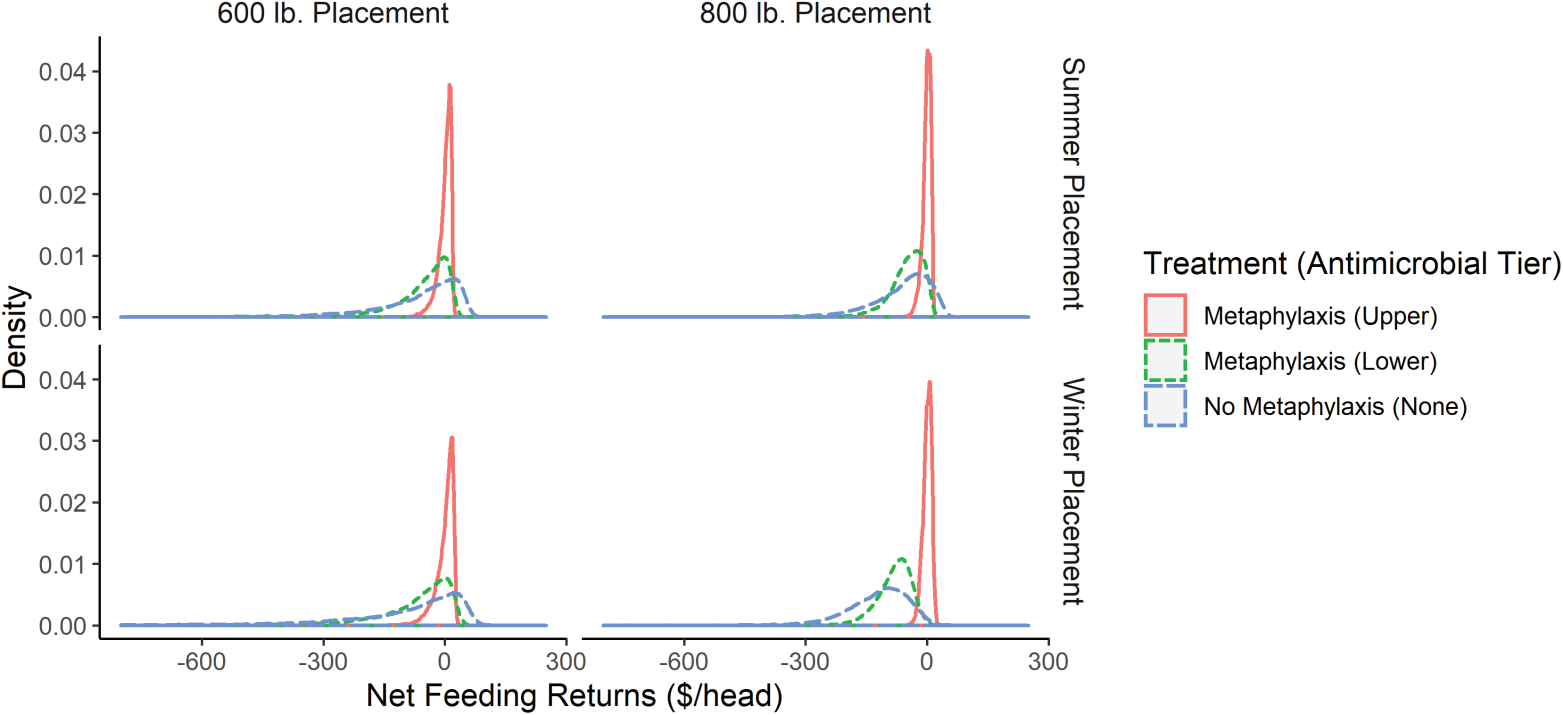
Net return distributions for high health-risk steers by placement weight, placement season, and type of antimicrobial used for metaphylaxis. Data used to generate the distributions are obtained from the simulation described in the main body of the text. Source: Author’s calculations.

Figure 2 displays net return distributions by cattle placement weight and season across three animal health management decisions: “no metaphylaxis,” “Upper Tier” antimicrobial, and “Lower Tier” antimicrobial. “No metaphylaxis” net return distributions represent high health-risk steer cohorts not managed with metaphylaxis upon arrival. The differences in expected net returns per head (averages) between administering an “Upper Tier” (“Lower Tier”) to “no metaphylaxis” were 1) $96.08 ($50.39) for 600 lb summer placements, 2) $122.55 ($65.72) for 600 lb winter placements, 3) $90.36 ($51.14) for 800 lb summer placements, and 4) $148.65 ($49.65) for 800 lb winter placements. The difference between the Upper Tier and Lower Tier is the marginal net benefit of using a certain antimicrobial tier for metaphylaxis. For example, the value of administering an “Upper Tier” compared to a “Lower Tier” for 600 lb summer placements is $45.69 (96.08−50.39 = 45.69). On average, “Upper Tier” antimicrobials were valued at $52.69 compared to “Lower Tier” antimicrobials across all placement weights and seasons.

Both antimicrobials used for metaphylaxis increased expected net returns and decreased net return variation. In other words, cattle feedlots were more certain that they will realize greater profits on a cohort of high health-risk cattle when any tier of antimicrobial was used for metaphylaxis. Table 5 further summarizes the net return distributions displayed in Figure 2 reporting percentages of the 5,000 simulated cohorts falling within expected net return ranges conditional on cattle placement weight, placement season, and antimicrobial used.

**Table 5.** Percentages of steer cohorts within net return ($/head) categories^a^

	600 lb Placement weight				800 lb Placement weight			
	Summer (Apr–Sept)		Winter (Oct–Mar)		Spring (Apr–Sept)		Winter (Oct–Mar)	
Net returns ($/head)	Upper	Lower	Upper	Lower	Upper	Lower	Upper	Lower
Metaphylaxis								
(-∞,-252)	0.00	1.64	0.00	4.40	0.00	0.04	0.00	0.04
(-251, -51)	1.64	37.56	3.64	40.32	0.08	41.72	0.00	76.64
(-50, 0)	34.12	40.56	34.44	33.12	41.68	51.68	47.80	23.00
(0, 50)	64.24	20.24	61.92	22.16	58.24	6.56	52.20	0.32
(>51)	0.00	0.00	0.00	0.00	0.00	0.00	0.00	0.00
No metaphylaxis								
(-∞,-252)	7.08	7.08	12.56	12.56	2.16	2.16	4.68	4.68
(-251, -51)	35.64	35.64	34.56	34.56	44.04	44.04	79.68	79.68
(-50, 0)	24.24	24.24	21.00	21.00	34.12	34.12	14.56	14.56
(0, 50)	33.04	33.04	28.16	28.16	19.68	19.68	1.08	1.08
(51, +∞)	0.00	0.00	3.72	3.72	0.00	0.00	0.00	0.00

^a^Antimicrobials used for metaphylaxis are categorized into “upper” and “lower” tiers based on Abell et al. (2017) who categorized them based on odds ratio (OR) confidence intervals.

Source: Author’s calculations.

Metaphylaxis administered to high health-risk cattle substantially reduced the probability of large losses. For example, for a cohort of 600 lb summer placed high health-risk steers treated with an “Upper Tier” antimicrobial, there was a 1.64% (0.00% + 1.64%) chance of realizing a loss of more than $50 per head; 0.00% probability of losses less than −$251 per head; 1.64% probability of losses between −$51 and −$251. If high health-risk steers were not treated, they faced a 42.72% (7.08% + 35.64%) probability of losing more than $50 per head. For a cohort of 600 lb high health-risk winter placed steers treated with a “Lower Tier” they faced a 44.72% (4.40% + 40.32%) chance of realizing at least a $50 per head loss. Not treating this same cohort resulted in a 47.12% (12.56% + 34.56%) probability of realizing at least a $50 per head loss. Taking this result further, large losses in excess of $250 per head had a 7% to 12% probability of being realized for high health-risk 600 lb placed steers not treated with any antimicrobial compared to generally less than 0% to 4% probability of such large losses regardless of season or antimicrobial tier used for metaphylaxis.

Heavier weight placements were less likely to have large negative returns since the proportion of cattle that died was relatively small compared to lighter placements. For example, a cohort of 600 lb summer placed high health-risk steers administered an “Upper Tier” antimicrobial faced a 1.64% (0.00% + 1.64%) chance of realizing a loss greater than $50 per head compared to 0.08% (0.00% + 0.08%) for a cohort of 800 lb steers placed at the same time and administered the same tier of antimicrobial. Summer placed high health-risk steers were expected to have a lower risk of large losses compared to winter placements. For example, a cohort of 800 lb summer placed high health-risk steers treated with a “Lower Tier” antimicrobial had a 41.76% (0.04% + 41.72%) probability of realizing a loss of more than $50 per head compared to 76.68% (0.04% + 76.64%) for the same cohort placed in winter.

## DISCUSSION

The objective of this study was to estimate net return and return risk between “Upper Tier” and “Lower Tier” antimicrobials used for metaphylaxis to manage BRD and a “no metaphylaxis” option in high health-risk feedlot cattle. We used a stochastic net return simulation model to determine how net return and return risk varied across cattle populations placed in different seasons and given different tiers of antimicrobials. As such, the outcomes are broad enough to encompass a variety of situations relevant to the feedlot industry.

The decision of whether to manage high health-risk cattle with metaphylaxis, and if so, which tier of antimicrobial to use, is a difficult question to answer in part because the decisions are made with incomplete information. Realized health outcomes are only known after cattle have finished feeding whereas metaphylaxis decisions are generally made at the time of cattle placement. Thus, we must rely on expected return distributions with and without the use of metaphylaxis to assess their expected economic value.

Metaphylaxis modifies cattle morbidity and mortality (Nickell and White, 2010; Tennant et al., 2014). In our model morbidity impacts were reflected through the decrease in cattle performance parameters (ADG, AFC, and HCs). Higher placement weights were associated with higher ADG and higher AFC consistent with randomized control trials that as placement weight increases so does AFC (Nickell and White, 2010). Positive correlations between HC and mortality are consistent with both trial and production-level data as both are associated with the rate at which feedlots are pulling and treating cattle. Estimated lower cattle performance in winter months is consistent with literature demonstrating cattle devote more energy to body temperature maintenance during colder months (Mader et al., 2010). “Upper Tier” antimicrobials are more expensive consistent with price premiums for a perceived higher quality product. Both antimicrobial tier cost estimates are generally consistent with results from the National Animal Health Monitoring Survey that reported average antimicrobial costs of $23.50 per head to administer metaphylaxis to feeder cattle (United States Department of Agriculture, 2013).

Mortality distributions were estimated as log-normal and fit using the mean and standard deviation of mortality for the different tiers of antimicrobials used for metaphylaxis and a “no metaphylaxis” option. Metaphylaxis modifies both the mean and standard deviation of mortality distributions consistent with previous literature (Dennis et al., 2018). Results also align with the knowledge that heavier cattle and summer placed cattle have lower mean mortality with less variation (Babcock et al., 2009). “Upper Tier” antimicrobials reduced both the variation and mean mortality values across all placement weights and placement seasons (Abell et al., 2017). Likewise, feedlots opting to use a “no metaphylaxis” option on high health-risk cattle significantly increases variation and mean mortality consistent with the idea that using any antimicrobial on high health-risk cattle is beneficial (Nickell and White, 2010).

An interesting result relative to the decision to manage high health-risk steer cohorts with metaphylaxis is that high net returns (low mortality) can still, by chance, be realized whether or not cattle are managed with metaphylaxis. For example, for 600 lb high risk steers placed, regardless of season or antimicrobial administered for metaphylaxis would expect more than 35% of the time to realize positive net returns. However, there is still at least a 20% chance that a high-risk cohort would realize positive net returns if not administered metaphylaxis. This is because there is a chance that high health-risk cattle, even if not given metaphylaxis, will remain sufficiently healthy and not have high mortality.

Expected return alone is insufficient to assess the viability of metaphylaxis if return risk also matters. Treating all high health-risk cattle with metaphylaxis broadly increases expected net return and reduces return variability. This makes the use of metaphylaxis as a health management practice appears obvious. However, the change in expected return, as well as the risk mitigated through metaphylaxis of all high-risk cattle, varies by placement season, tier of antimicrobial used, and cattle placement weight. Lighter weight high health-risk cattle are expected to realize greater returns and more return risk mitigation through metaphylaxis regardless of the season and antimicrobial tier (of those investigated here), than heavier weight placements.

The value of meeting production or marketing contract agreements is a value of metaphylaxis not currently captured in the simulation. Feedlots can use metaphylaxis as a preventative measure to help enable them to have enough cattle reach harvest weight to comply with agreements/contracts with meat packers. Thus, the value of metaphylaxis represents more than just the loss in input costs as currently calculated. Thus, estimated values likely serve as a lower bound to the value of metaphylaxis for feedlots.

Our simulation had feedlots sell cattle on a live weight basis and did not allow for any discounts or premiums for carcass yield and quality grade. Thus, if carcass quality is affected by the use of metaphylaxis then the net value of metaphylaxis would change based on premiums and/or discounts for yield and quality grade.

An important issue, not addressed in this study but deserves more consideration given our findings, is how to identify and categorize high health-risk cattle. The occurrence of respiratory disease in some cattle in a cohort, BRD problems in cattle previously received from the same source, and known lack of preconditioning are all important in determining whether a group of cattle should be managed with metaphylaxis (United States Department of Agriculture, 2019). Feedlots can discount high health-risk cattle at purchase to offset perceived production and HCs. High-risk cattle are worthless to the feedlot than otherwise similar low-risk cattle, since they can require higher HCs and have greater morbidity and mortality risk with more variable net returns. Feedlots that are able to correctly categorize cattle health risk could capitalize on these discounts.

In practice high health-risk cattle are managed with metaphylaxis and feedlots do not observe net return outcomes from high health-risk cattle not managed with metaphylaxis. While positive profits can be obtained, feedlots may perceive the realization of major net return losses to be worse than the potential positive profits from not managing with metaphylaxis. Likewise, the error associated with mis-categorization of cattle into health-risk bins further complicates this decision process. We expect with the more accurate categorization of cattle into health-risk status upon feedlot arrival, health management strategies could be refined. Of course, the cost of acquiring additional information for more accurate animal health status classification may exceed the value. For example, chute-side diagnostics aimed at identifying sick cattle on arrival may be too costly or time and labor prohibitive.

The estimates from our study should be held in the context of the fed and feeder cattle price levels used. Previous work indicates that changing input and output price levels can impact the value of metaphylaxis (Dennis et al., 2018) and in our case the value for each antimicrobial tier. The average fed cattle price used in this simulation was $148/cwt (see Table 1). Higher fed cattle prices create greater value associated with metaphylaxis, all else held equal. As fed cattle prices increase, the cost of mortality increases. As such, fed cattle price has important impacts on the value of metaphylaxis because higher fed cattle prices are associated with higher feeder cattle prices and any mortality has a greater economic cost to the feedlot.

## CONCLUSIONS

Results identified the net return and return risk of using two different antimicrobial tiers for metaphylaxis compared to no metaphylaxis, in different high-risk cattle populations. Likewise, we quantified the relative benefit of using an “Upper Tier” antimicrobial compared to a “Lower Tier” antimicrobial for metaphylaxis. “Upper Tier” antimicrobial produced higher net returns and lower return risk compared to “Lower Tier” antimicrobials. Both antimicrobial tiers were valued more than not administering metaphylaxis, but this expected value varies by cattle placement season and placement weight. Further research is needed to determine how feedlots substitute between these tiers of antimicrobials in an attempt to match animal health risk with antimicrobial tier effectiveness and cost in an effort to maximize profit.
